# Molecular Mechanism of Action of Low-Intensity Extracorporeal Shockwave Therapy for Regenerating Penile and Peripheral Nerves

**DOI:** 10.5152/tud.2022.20419

**Published:** 2020-10-09

**Authors:** Dongyi Peng, Yan Tan, Amanda B. Reed-Maldonado, Guiting Lin, Tom F. Lue

**Affiliations:** 1 Knuppe Molecular Urology Laboratory, Department of Urology, School of Medicine, University of California, San Francisco, USA; 2 Department of Urology, The Third Xiangya Hospital of Central South University, Changsha, China

**Keywords:** Activation, cellular signaling, low-intensity extracorporeal shockwave therapy, neurotrophic factors, peripheral nerve regeneration, Schwann cells

## Abstract

Sufficient functional repair of damaged peripheral nerves is a big clinical challenge in terms of long-lasting morbidity, disability, and economic costs. Nerve damage after radical prostatectomy is the most common cause of erectile dysfunction. In recent years, low-intensity extracorporeal shockwave therapy has been explored to improve the outcomes of peripheral nerve repair and regeneration. Research indicated that application of low-intensity extracorporeal shockwave therapy after nerve surgery promoted nerve regeneration and improved the functional outcomes, underlined the mechanisms related to increase of neurotrophic factors, Schwann cells activation, and cellular signaling activation for cell activation and mitosis induced by low-intensity extracorporeal shockwave therapy. We searched PubMed for articles related to research on these topics in both *in vitro* and *in vivo* animal models and found numerous studies suggesting that the application low-intensity extracorporeal shockwave therapy could be a novel treatment for erectile dysfunction induced by nerve injury and other disease related to nerve injury.

**Main Points**

- Low-intensity extracorporeal shockwave therapy improved peripheral nerve repair and regeneration.
- Low-intensity extracorporeal shockwave therapy exerts its biological effects by increasing neurotrophic factors, Schwann cell activation, and cellular signaling activation.
- Low-intensity extracorporeal shockwave therapy could be a novel treatment for nerve injury-induced erectile dysfunction and other conditions related to nerve injury.

## Introduction

The efficient functional repair of damaged peripheral nerves is a big clinical challenge because they are vulnerable to injuries from crushing, stretching, compression, and avulsion and may result in long-lasting morbidity, disability, and economic costs.^1-3^ The causes of peripheral nerve injury could be traffic accidents, tumor damage, viral infections, side effects of neurosurgery, and so on.^4^ Injuries to the peripheral nerves can also occur in multiple clinical scenarios. For example, prostate cancer surgery often damages the corpus cavernous nerve, even with nerve-sparing techniques,^5^ which eventually leads to erectile dysfunction (ED). Radical prostatectomy is the gold standard for early-stage prostate cancer but is also the most common cause of ED. The prevalence of ED is approximately 14%-90% because of nerve damage after radical prostatectomy.^6^

As the peripheral nervous system is capable of regeneration, injuries are usually reconstructed by primary repair. However, multiple difficulties exist with the process of regeneration over long distances, such as following proximal lesions or nerve gaps. In these instances, the injury repair needs artificial conduits or the gold standard autologous nerve grafts. The nerve gap and slow axonal regeneration present a limiting factor for efficient reinnervation.^7-10^ However, the treatment of nerve injury after radical prostatectomy is still limited,^11^ and the prognosis is poor if treatment is delayed because the functioning nerves are necessary for erections. One of the approaches to accelerate peripheral nerve regeneration is to stimulate the physiological processes that occur after nerve injury.

As slow axonal regeneration is the unsolved key issue limiting the functional outcome after nerve surgery, many methods, including various forms of external physical stimulation (electric stimulation,^12^ laser stimulation,^13^ magnetic field,^14^ and so on) and biological therapy (administration of neurotrophic factors,^15^ vitamins,^16^ and medications^17^), have been proved to enhance the nerve regeneration although there are some limitations to their clinical application, and a novel and effective therapeutic approach to stimulate the physiological processes is needed.

Recently, low-intensity extracorporeal shockwave therapy (LiESWT) has been successfully used in the field of regenerative medicine after its original introduction as urological lithotripsy.^18^ In preclinical and clinical trials, Li-ESWT is currently applied to a wide range of medical indications, such as wound healing,^19^ musculoskeletal disorders,^20^ bone healing disturbances,^21,22^ painful scars,^23^ spastic hypertonia,^24^ ischemic heart diseases,^25^, and so on. More recently, studies focusing on the influence of ESWT on peripheral nerve proved that Li-ESWT could promote peripheral nerve regeneration after injury.^26,27^ Although no clinical studies exist regarding the same, several experimental studies have investigated the use of Li-ESWT as an effective treatment after peripheral nerve repair and demonstrated very good outcomes. This study presents a systematic review of the available preclinical literature of the reported effects of Li-ESWT in penile and peripheral nerve regeneration and its potential clinical applications.

## Pathogenesis of Nerve Injury and Regeneration

Peripheral nerves are particularly vulnerable to injuries, and the peripheral nervous system has the ability to regenerate in contrast to the central nervous system. The pathophysiology of peripheral nerve injuries and the mechanisms involved in spontaneous regeneration are relatively well understood, and there is some evidence that a conditioning lesion primes the peripheral nerve for regeneration;^28^ however, the functional recovery is often incomplete.

The process of spontaneous regeneration starts with the initial response to injury, such as after complete nerve transection.^29^ After nerve transection, the distal nerve ending undergoes Wallerian degeneration, which is a unique and structured form of axon degeneration.^30^ At first, axonal and myelin debris are produced, and resident macrophages in the nerve tissue then differentiate into activated macrophages to phagocytose the cellular debris. Activation of messenger-ribonucleic acid translation (mRNA) is observed in the proximal stumps in the axons, which stimulates the formation of the protein complex importin-phosphorylated extracellular regulated protein kinase 1/2 vimentin. This complex is transported by the motor protein dynein in a retrograde direction to the cell body, and this signal informs the neuron of the axonal damage.^31^ The neuron of soma then reacts by breaking up Nissl bodies which promotes protein synthesis and peripheral displacement.^29,32^ Only a few hours after the nerve injury, the growing axonal extremity extends filopodia, which are randomly oriented at first but gain unidirectionality thereafter, and the proximal stump sprouts processes that sample the environment for neurotrophic factors to guide them to their target.^33-35^

Successful peripheral nerve regeneration after injury relies on both injured axons and non-neuronal cells, including Schwann cells (SCs), endoneurial fibroblasts, and macrophages, which produce a supportive microenvironment for allowing successful regrowth of the proximal nerve fiber ending.^36^ Schwann cells play an important role in the axonal regeneration and can secrete chemokines, such as monocyte chemoattractant protein-1, which leads to the recruitment of circulating macrophages for the removal of myelin and axonal debris.^37,38^ Schwann cells produce neurite-promoting proteins, such as fibronectin, laminin, tenascin, heparin sulfate, and collagen, which are incorporated into the extracellular matrix that is lost because of injury.^39^ The proliferating SCs are aligned in columns forming “bands of Büngner” that form a physical guide for new axonal regrowth.^40,41^ Schwann cells express cell adhesion molecules that are important in interacting with matrix proteins that will modulate the axon outgrowth and path finding.^39,42,43^ Schwann cells also express neurotrophic factors, such as ciliary neurotrophic factor, brain-derived neurotrophic factor, glial cell line-derived neurotrophic factor, and nerve growth factor, which can increase the cell survival and promote nerve regeneration.^36^ Recently, it was reported that SCs regulate peripheral nerve regeneration by secreting exosomes.^44^

## Physical Characteristics of Shockwave

The shockwave is defined as a sonic pulse, initially spiking to a high peak pressure of up to 100 MPa in 10 ns and then falling to a negative pressure of about 5-10 MPa duration up to 5 μs, thought to induce biological reaction to the targeted tissues by the high initial pressure, and proceeded by a tensile force and mechanical stimulation.^45^ According to the energy level, the ESWT can be divided into high-intensity ESWT (Hi-ESWT) and Li-ESWT energy categories. Although both treatment modalities are therapeutic, the Hi-ESWT is typically administered for destruction of solid aggregations inside or outside tissues,^46,47^ whereas Li-ESWT treatment is used for tissue repair and regeneration.^48^

The history of shockwaves as a therapeutic approach is relatively short. In the 1980s,^49^ shockwave was first used for destruction of kidney and urinary stones. In recent years, Li-ESWT has become a widely utilized therapeutic tool in regenerative medicine. The positive effects of Li-ESWT on peripheral nerve regeneration have also been reported recently,^50,51^ whereas the Hi-ESWT may cause myelin sheath damage histologically and further functional damage in horses and dogs.^52,53^

In the field of ED treatment, Li-ESWT has shown positive therapeutic effect mainly in non-neurogenic ED in the recent years,^54-57^ but the applicability of Li-ESWT in neurogenic ED, such as that occurring in postradical prostatectomy (post-RP ED) because of nerve damage, is questionable.^55^ However, some studies showed that Li-ESWT might increase the rate of blood flow and regenerate the nervous tissue when applied to penile tissue^58^ and can provide a positive effect to neurogenic ED with nerve damage, such as post-RP ED.^59,60^ However, the potential mechanisms producing these biological effects are still unclear and need further investigation.

## Effect of Extracorporeal Shockwave Therapy on Peripheral Nerve Regeneration

As mentioned earlier, ESWT with different levels of energy has different therapeutic effects. Some studies have found that ESWT could cause damage to the peripheral nerve, and the safety of ESWT was therefore challenged. In 2002, Wang et al^52^ found that Hi-ESWT (0.47 mJ/mm^2^) can cause injury to the nerve and lead to mild nerve bundle swelling in a dog’s femoral nerve. Wu et al^61^ used the sciatic nerve of rats to investigate the effects of varying intensities of ESWT on the peripheral nerve and found moderate decrease in the motor nerve conduction velocity and damage to the myelin sheath of the large-diameter myelinated fibers after all levels of intensity of ESWT were applied. The effect was larger and longer in duration in the highintensity group, and all the changes were reversible.

Overall, beside the therapeutic effect, there are some evidences to prove that ESWT can cause reversible damage to the peripheral nerve in an intensity-dependent manner and that the LiESWT is a safe method to treat nerve injury. Thus, in this review, we focused on the effect of Li-ESWT on peripheral nerve regeneration.

## Dosage Effect of Low-Intensity Extracorporeal Shockwave Therapy on the Peripheral Nerve

Evidence suggests that Li-ESWT less than 900 pulses combined with a flux density of 0.08 mJ/mm^2^ should be safe, and Li-ESWT more than 900 pulses could induce damage to the peripheral nerves.

In 2001, Ohtori et al^62^ found that 1000 pulses of shockwaves (0.08 mJ/mm^2^, 2.4 Hz) can cause degeneration of the sensory nerve fibers and endings followed by reinnervation of the affected skin areas. In 2006, Takahashi et al^63^ found that a second application of the same dose of Li-ESWT had a cumulative effect on the treated nerves, leading to delayed reinnervation, which can be reversed within 2 weeks. In 2008, Wu et al^61,64^ manifested that application of 2000 pulses of Li-ESWT (0.08 mJ/mm^2^) impaired the electrophysiological conduction parameters in the sciatic nerve of rats, which could be reversed in 1 week. In 2012, Hausner et al^26^ found that the sciatic nerves of rats treated with different dosages of Li-ESWT (0.1 mJ/mm^2^) have different effects. The result showed that 300 pulses did not induce axonal degeneration 1 week after ESWT, whereas treatment with 900 and 1500 pulses resulted in moderate and severe degeneration, respectively. Although Li-ESWT is safer than HiESWT, to treat nerves, the dosage should be controlled to avoid damage to the nerves.

## Low-Intensity Extracorporeal Shockwave Therapy Promotes Peripheral Nerve Regeneration

Despite these well-known effects of Li-ESWT on many kinds of cells and tissues, including peripheral nerves, little was known about its effects on either intact or damaged nerve tissue, which represents the effects on nerve regeneration till very recently. There are some studies investigating whether and how Li-ESWT influences the regeneration of damaged peripheral nerves.

In 2012, Hausner et al^26^ used rats’ sciatic nerve defect model with an 8-mm long right side sciatic nerve reversed homotopic autologous nerve transplantation to explore the effect of Li-ESWT (3 Hz, 0.1 mJ/mm^2^, 300 pulses) on nerve regeneration. They found that 3 weeks after surgery, the morphological data presented faster elongation of the myelinated axons and far more regenerating myelinated fibers in the Li-ESWT nerves than in the control nerves. The morphological improvement correlated with the electrophysiological result that nerve action potentials with considerable amplitudes could be evoked at 3 weeks in the sciatic nerve of the animals treated with Li-ESWT but not in the nerves of the control animals. After the regenerating nerves reached their peripheral targets, such as skeletal muscles, they reinnervated the targets and then produced a functional reinnervation at 6 to 8 weeks after surgery. Overall, the results showed that Li-ESWT might improve the functional recovery in the initial phase of regeneration after the sciatic nerve injury in rats. They also assumed that Li-ESWT improved reorganization of the injured nerves owing to faster clearance, fewer fibroblasts, and less endoneural collagen, which provided a lower degree of endoneural scarring and fibrocytic activity.

In 2013, Lee and Cho^65^ used rats’ sciatic nerve-crushing damage model to explore the effect of Li-ESWT (3 Hz, 0.09 mJ/mm^2^, 300 pulses) on muscle weight and function. They found that 14 days after surgery, the Li-ESWT group showed a significant increase in the sciatic functional index score and reduced level of muscle atrophy compared with those of the control group. According to their results, they assumed that although Li-ESWT stimulates regeneration and reordering of the injured nerves, activates the conjunction of the muscle and neurons, and increases the functional activity, it also counteracts the changes in the nerve damage, including the inhibition of muscle contraction and decrease of protein synthesis to reduce muscle atrophy.

In 2015, Lee and Kim^51^ used a rat model to explore the effect of Li-ESWT (3 Hz, 0.09 mJ/mm^2^, 300 pulses) on functional recovery and neurotrophin-3 (NT-3) expression in the spinal cord after sciatic nerve-crushing damage. They found that Li-ESWT can promote the expression of NT-3 compared with the control group, which could facilitate the activity of macrophages and SCs, which affects the survival and regeneration of neurons. This, finally, resulted in a continuous and statistically significant increase in the functional activity in the Li-ESWT group compared with that of the control group.

## Effect of Low-Intensity Extracorporeal Shockwave Therapy on Neurogenic Erectile Dysfunction

In recent years, the therapeutic effect of Li-ESWT has been studied mainly in non-neurogenic ED, and the effect is primarily related to the stimulation of cell proliferation, tissue regeneration, and angiogenesis.^54-57^ However, studies have also investigated the effect of Li-ESWT in the treatment of neurogenic ED with nerve damage, such as post-RP ED, and showed considerable application prospects.^59,60^

In 2016, a pilot study by Frey et al^60^ included 16 patients who had undergone robot-assisted bilateral nerve-sparing RP and suffered from mild to severe postoperative ED for more than a year. They received 2 Li-ESWT sessions every alternative week for 6 weeks. Each treatment session included 3000 shockwaves with a frequency of 5 Hz at different energy densities, and the shockwaves were applied to the root of the penis, to the shaft, and at a few millimeters proximal to the glans. They found that Li-ESWT ameliorated the erectile function with median improvement to the 5-item International Index of Erectile Function scores significantly at 1 month and 1 year after treatment. However, the improvements did not allow for unassisted erections sufficient for intercourse in most patients.

In 2016, Li et al^66^ developed a rat ED model related to pelvic neurovascular injuries to investigate the therapeutic effect of Li-ESWT (0.06 mJ/mm^2^, 300 pulses, 3 Hz) on neurogenic ED. The pelvic neurovascular injury model was established by bilateral cavernous nerve injury and internal pudendal bundle injury (PVNI). They found that Li-ESWT could significantly promote the erectile function and major penile nerve regeneration, including neuronal nitric oxide synthase (nNOS) nerve fibers after PVNI, compared with those in the control group. In their experiment, they also found that Li-ESWT can promote the Schwann dedifferentiation and proliferation, which result in more mature SCs and good environment amenable to nerve regrowth. Therefore, they assumed that Li-ESWT had a therapeutic effect on neurogenic ED through activation of SCs, promoting nerve regeneration.

## Low-Intensity Extracorporeal Shockwave Therapy Activates Schwann Cells

As SCs play a predominant role in the process of peripheral nerve regeneration,^67-70^ some studies focused on the effects of Li-ESWT on SCs both *in vivo* and *in vitro*. In the *in vivo* study, Li et al^66^ used a rat ED model related to pelvic neurovascular injuries to investigate the effect of Li-ESWT (0.06 mJ/mm^2^, 300 pulses, 3 Hz) on the activation of SCs. Using the Western blotting technique, they found that the expression of p75 and p-Erk1/2 significantly increased in the penile tissue after Li-ESWT. This indicated that Li-ESWT could activate extracellular signal-regulated kinase (ERK)/mitogen-activated protein kinase (MAPK) and p75 to induce SC dedifferentiation and proliferation in the damaged nerves. Furthermore, there were more mature SCs (S100 positive SCs) in the damaged dorsal nerves in the Li-ESWT group than in the control group by immunofluorescence staining. They assumed that Li-ESWT could stimulate dedifferentiation and proliferation of SCs in the damaged nerve by activation of ERK/MAPK and p75, which resulted in more mature SCs to promote nerve regeneration.

As activation of SCs by Li-ESWT *in vivo* is considered one of the possible mechanisms to promote nerve regeneration, there are numerous studies investigating the effect of Li-ESWT on SCs *in vitro*. In 2016, Schuh et al^71^ used rat sciatic nerves to elucidate the effects of Li-ESWT (0.10 mJ/mm^2^, 300 pulses, 3 Hz) on SC isolation and culture. After dissection, the sciatic nerves were treated with Li-ESWT, and the SCs were isolated and cultured for 15 passages. The result showed that the quality of the cultured SCs, including the purity, proliferation rate, and expression of regenerative–phenotype-associated markers, was significantly improved in the Li-ESWT group. In contrast, the control group exhibited progressively senescent behavior, such as decrease in proliferation, loss of specific markers, and increase in P16^INK4A^ expression. In 2016, Li et al^66^ used Li-ESWT to culture adherent rat SCs. Their result showed that the expressions of p-Erk1/2 and p75 were significantly elevated using Western blot, and p-Erk1/2 tended to accumulate in the SC nuclei in immunofluorescence staining, which indicated that Li-ESWT triggers the initiation of p-ERK1/2-mediated downstream pathways in SCs. In addition, they found that a higher percentage of SCs entered the S phase and G2/M when treated with Li-ESWT than the untreated cells. Overall, these data demonstrate the growth-promoting effect of Li-ESWT on SCs. In 2017, Wang et al^72^ treated RT4-D6P2T (rat SCs) with Li-ESWT (0.01 mJ/mm^2^, 3 Hz, different pulses) and found that Li-ESWT activated the protein kinase RNA-like endoplasmic reticulum (ER) kinase (PERK) pathway and enhanced the activating transcription factor 4 (ATF4) in an energy-dependent manner, which resulted in the increased expression of brain-derived neurotrophic factor (BDNF), which could benefit nerve regeneration.

Hence, multiple evidences exist to prove that Li-ESWT can activate and promote SC proliferation, both *in vivo* and *in vitro*, which should be of great benefit for nerve regeneration. This may be one of mechanisms through which Li-ESWT promotes peripheral nerve regeneration after injury.

## Low-Intensity Extracorporeal Shockwave Therapy Induces Neurotrophic Factors

Neurotrophic factors (NFs) are a class of secreted proteins, which are essential during the development and differentiation of the central and peripheral nervous system. Neurotrophic factors include nerve growth factor (NGF), BDNF, NT3, and so on.^73^ Since their discovery in the 1950s by Levi-Montalcini and Hamburger,^74^ *in vitro* and *in vivo* animal experiments have elucidated their strong ability to elicit positive survival and functional responses in the neurons of the peripheral and central nervous system.^73^ After nerve injury, NFs are essential in controlling the survival, proliferation, and differentiation of neural and nonneural cells involved in nerve regeneration.^14,27^

## Brain-Derived Neurotrophic Factor

Brain-derived neurotrophic factor, as a member of the NF family, plays an important role in the survival of the existing neurons and the differentiation of new neurons.^75^ It is associated with axonal regeneration, myelinogenesis of the medullated nerve fibers,^76^ and SC regeneration^77^ during the repair of nerve injury and is thus a promising therapeutic molecule. In ED, BDNF has been demonstrated to enhance the regeneration of nNOS and recovery of erectile function.^78,79^ In 2017, Wang et al^72^ found that Li-ESWT could stimulate the expression of BDNF both *in vivo* and *in vitro*. For the *in vivo* demonstration, they treated bilateral cavernous nerve crush injury (BCNI) in rats with Li-ESWT (0.06 mJ/mm^2^, 3 Hz, 500 pulses) twice in a week and found that Li-ESWT significantly promoted the expression of BDNF in penile tissues at RNA level. With the use of Li-ESWT, the expression levels of BDNF in the penis increased 3 days after injury and remained at a stable level for up to 26 days. For *in vitro* demonstration, they treated RT4-D6P2T (rat Schwann) cells with Li-ESWT (0.01 mJ/mm^2^, 3 Hz, different pulses) and found that Li-ESWT increased the expression of BDNF at the RNA level. Furthermore, the Western blot result also indicated that Li-ESWT increased BDNF through activation of PERK/ATF4 signaling pathway. Therefore, Li-ESWT could promote BDNF secretion both *in vivo* and *in vitro*, and the increase in BDNF may benefit nerve regeneration after nerve injury and the treatment of neurogenic ED.

## Neurotrophin-3

Neurotrophin-3 is a key NF constituent in the peripheral nervous system as an important regulator of the neural survival, development, function, and neuronal differentiation.^80^ At the same time, NT-3 is an important autocrine factor, supporting SC survival and differentiation in the absence of axons.^81^ Neurotrophin-3 also has an i mportant role in the axonal extension, survival and maintenance of neurons, and myelination and regeneration of neural fibers in nervous injury.^82^ In 2015, Lee and Kim^51^ used a rat model to explore the effect of Li-ESWT (3 Hz, 0.09 mJ/ mm^2^, 300 pulses) on NT-3 expression in the spinal cord after sciatic nerve-crushing damage. Low-Intensity Extracorporeal Shockwave Therapy significantly increased the expression of NT-3 1 day after nerve crushing and remained at a stable level for up to 14 days compared with the levels in the sham and control groups. They assumed that the application of Li-ESWT increased the expression of NT-3, which facilitated the activity of macrophages and SCs, which promoted the survival and regeneration of the neurons.

## Effect of Low-Intensity Extracorporeal Shockwave Therapy on Cellular Signaling for Cell Activation and Mitosis

Low-Intensity Extracorporeal Shockwave Therapy is a mechanical force that can stimulate the tissues, especially cells. The conversion of mechanical force into biochemical signals is referred to as mechanotransduction. Although the mechanism of Li-ESWT-induced mechanotransduction in target cells is still not clear, different pathways of biological reactions that derive from these mechanical forces were studied recently. There are various mechanisms behind the effects in nerve regeneration after Li-ESWT.

## Extracellular Signal-Regulated Kinase Pathway

In 2014, Weihs et al^83^ elucidated in their study that ESWT could activate adenosine triphosphate (ATP) release-coupled ERK pathway in several cell types (e.g., mesenchymal stem cells) to stimulate cell proliferation. In 2016, Schuh et al^71^ applied Li-ESWT to the whole sciatic nerve before isolation of SCs and found that it could enhance the extracellular levels of ATP. Adenosine triphosphate can activate purinergic metabotropic P2Y receptors, then downstream the Erk1/2 signaling, and finally enhance cell proliferation. In 2016, Li et al^66^ also found that Li-ESWT could activate Erk1/2 in the rat SCs. Their result showed that the expression of p-Erk1/2 was significantly elevated at the protein level, and p-Erk1/2 tended to accumulate in the SC nuclei in immunofluorescence staining. In 2016, Zhao et al^84^ found that the activation of ERK1/2 in cultured PC12 cells could phosphorylate cyclic adenosine monophosphate response element-binding protein (CREB) and promote the expression of thioredoxin-1 (Trx-1). Thioredoxin-1 has various biological activities, including antioxidant effects, neurotrophic cofactor, cell growth promotion, and cellular apoptosis suppression.

## Protein Kinase RNA-Like Endoplasmic Reticulum Kinase Pathway

In 2017, Wang et al^73^ found that Li-ESWT activated the PERK pathway and enhanced ATF4, which resulted in the increased expression of BDNF in the rat SCs. Protein Kinase RNA-Like Endoplasmic Reticulum Kinase/ATF4 pathway, a mechanistic branch of the unfolded protein response, is responsible for the attenuation of the overload of misfolded proteins, thereby alleviating the ER stress. In their study, they found that Li-ESWT activated the PERK pathway by increasing the phosphorylation level of PERK and eukaryotic initiation factor 2α and enhanced ATF4 expression in an energy-dependent manner. This resulted in the increased expression of BDNF.

## Tropomyosin Receptor Kinase B Pathway

As mentioned earlier, Li-ESWT can promote the expression of BDNF and NT-3, which can mediate their effects through their high affinity for the tropomyosin receptor kinase B (TrkB) receptor. In 2017, Su et al^85^ found that increased BDNF proteins activated TrkB and triggered the downstream phosphatidylinositol 3-kinase/protein kinase B signaling pathway and increased the phosphorylation of CREB.^86^

**Figure 1.**
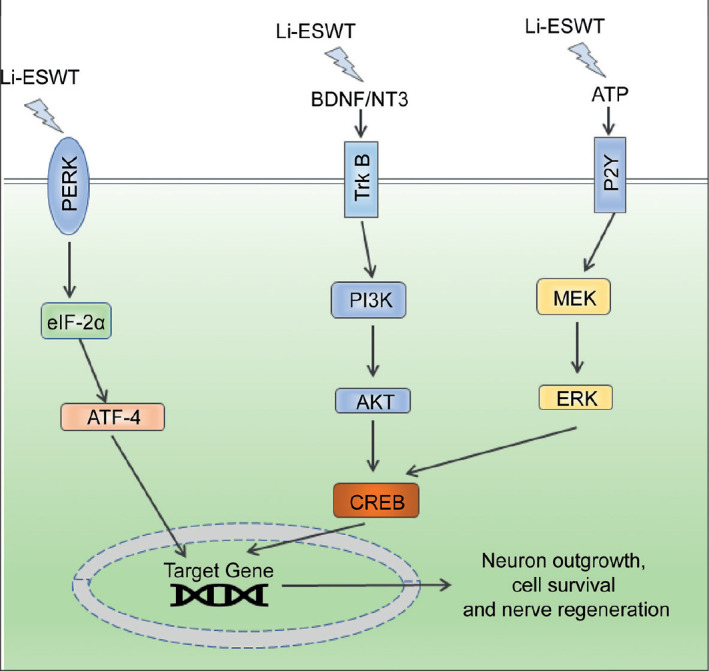
Cellular Signaling Pathways Regulated by LowIntensity Extracorporeal Shockwave Therapy for Peripheral Nerve Regeneration.

In conclusion, there is a significant evidence to prove that the application of Li-ESWT after nerve surgery promotes nerve regeneration and improves the functional outcomes. The benefits of Li-ESWT in peripheral nerve regeneration and neurogenic ED may be owing to the increase in NFs, SC activation, and cellular signaling activation for cell activation and mitosis (Figure 1). Given the preclinical benefits in the absence of any negative side effects, Li-ESWT should be investigated clinically in humans as an adjunct therapy after nerve surgery.
